# Being a “Warrior” to Care for the New Family: A Meta-ethnography of Nurses’ Perspectives on Municipal Postnatal Healthcare

**DOI:** 10.1177/23333936231218843

**Published:** 2023-12-25

**Authors:** Bente Kristin Høgmo, Marit Alstveit, Terese Bondas

**Affiliations:** 1 University of Stavanger, Norway

**Keywords:** postnatal health care, perspectives, caring, nurse, meta-ethnography

## Abstract

Care in the postnatal period is a goal for all families with a newborn baby, and support from nurses might prevent long-term health problems and contribute to a positive postnatal experience. This meta-ethnography aims to integrate and synthesize qualitative studies that illuminate and describe nurses’ perspectives on municipal postnatal health care in high-income countries. Systematic literature searches for qualitative studies were conducted and 13 articles were included. The analysis followed the seven phases of Noblit and Hare. *Being a “warrior” to care for the new family* was identified as an overarching metaphor accompanied by three main themes: *Stretching human boundaries, Stretching system boundaries*, and *Stretching knowledge boundaries.* The overarching metaphor offers a deeper understanding of the nurses as “warriors” who despite tight timeframes and heavy workloads are stretching toward a caring relationship with the families. Being a warrior continuously pushing system boundaries puts the nurses in risk of being overstretched, balancing between their ideals and the reality. As more knowledge and clearer policies and procedures regarding the inclusion of fathers and LGBTQ parents in municipal postnatal healthcare are needed, more focus placed on the father or non-birthing parent, different cultural traditions and family constellations in practice and education is suggested.

## Introduction

The postnatal period is the phase beginning immediately after the birth of the baby and extending for up to 6 weeks after birth (World Health Organization [WHO], 2018). An early discharge of mother and newborn from hospital after birth has become common in many western countries, and this marks a shift from an illness orientation to a more family-centered approach to maternity care (Campbell et al., 2016; Jones et al., 2021). Worldwide, it is a goal that new parents should receive support, reassurance, and information during the postnatal period (WHO, 2022). Home visits by nurses are important, as they may prevent long-term health problems effecting women, their babies, and families (Yonemoto et al., 2021). Through early contact with the mother and baby, nurses are given an opportunity to facilitate breastfeeding, monitor the overall health and growth of the baby, screen for parental postpartum depression, treat childbirth-related complications and refer the mother and newborn for specialized care, if necessary (Finlayson et al., 2020; WHO, 2022). While the WHO (2022) guideline states that it aims to improve the quality of essential, routine postnatal care for women and newborns with the goal of improving maternal and newborn health and well-being, it is recognized that good quality postnatal care to the family needs to be inclusive of the father or non-birthing parent (Barimani & Vikström, 2015; Entsieh & Hallström, 2016; Wiklund et al., 2018). Good parental support is linked to receiving sufficient information, parent participation and individually adapted care.

Although the importance of good quality postnatal care is well recognized, the proportion of babies and parents who receive postnatal care is significantly lower than those who receive antenatal care, which constitutes a missed opportunity for the new family (WHO, 2022). A reason for this could be that postnatal care is not accessible and/or not of good quality. It could also reflect that the focus has mainly been on the number of visits and timing of the postnatal care rather than on the content and care provided (McCauley et al., 2022).

Postnatal care services are key to achieving the Sustainable Development Goals (SDGs) on reproductive, maternal and child health (WHO, 2022). In line with the SDGs, and in accordance with a human rights-based approach, postnatal care efforts must expand beyond survival and coverage alone to include quality of care. To reach the goal of a positive postnatal experience, qualified and motivated nurses and resourced and flexible health systems are needed. Globally, there is a diversity of health professionals involved in providing care and support during the postnatal period, including midwives, nurses, community health workers, general medical practitioners, pediatricians, etc. (WHO, 2022). Public health nurses (PHN) and midwives are often key health professionals in municipal postnatal health care who support families with newborn babies (Glavin & Leahy-Warren, 2013), although the terminology, range and scope of public health nursing practices varies between different countries (Noonan et al., 2017). In European countries, the titles “PHN” or “health visitor” are used, while, for example, in Australia, “Maternal, Child, and Family Health Nurse” is used. In this meta-ethnography, we have used the term “nurses” when referring to health professionals offering postnatal care in the community setting.

## Background

The postnatal period is often described as a stressful time in the life of parents, with an increased need for both information, clinical care and psychosocial- and emotional support in order to feel secure in the parenting role (Høgmo et al., 2023; Wiklund et al., 2018). Achieving positive motherhood, fulfilling adaptation to changed intimate and family relationships, and regaining health and wellbeing after giving birth are important for new mothers (Finlayson et al., 2020). Research on nurses’ perception of mothers’ postnatal needs shows that it appears to be a discrepancy of opinion between professionals and mothers about care needs in the postnatal period (Slomian et al., 2017). The professionals were more concerned about mothers’ needs during pregnancy than those in the postnatal period, perceiving that mothers were well informed about the time after birth and just needed time to assimilate it. The mothers, on the other hand, described being well prepared and cared for during pregnancy and birth, but after the delivery, it was described as “there is nothing.” Postnatal care has historically focused almost solely on maternal and infant wellbeing (Eberhard-Gran et al., 2010; WHO, 2014), but today most mothers and fathers see parenting as a joint project which requires equal opportunities and support from the healthcare system (Høgmo et al., 2021; Wiklund et al., 2018). Studies show that both mothers and fathers have support needs during their transition to parenthood (Hrybanova et al., 2019; Sacks et al., 2022; Shorey et al., 2017). Nurses play an important role in identifying, supporting, and caring for mothers with postpartum depression (Alexandrou et al., 2018; Ashford et al., 2017; Vik et al., 2021) and how new parents experience and manage the initial postnatal period can impact the well-being of the entire family (Baldwin et al., 2018; Delicate et al., 2018; Solberg et al., 2023). As depression in fathers also is a serious mental health concern in the transition to parenthood (Cameron et al., 2016), implementing The Edinburgh Postnatal Depression Scale (EPDS) in postnatal care for both parents is suggested (Asper et al., 2018; Carlberg et al., 2018; Shafian et al., 2022).

Studies on lesbian, gay, bisexual, transgender or queer (LGBTQ) parents describe both positive and negative experiences in the encounter with health professionals during their transition to parenthood, revealing that heteronormativity and a lack of LGBTQ competence might lead to a lack of support when these individuals become parents (Andersen et al., 2017; Klittmark et al., 2019).

## Theoretical Perspective

The theory of caritative caring (Bergbom, Nåden, & Nyström, 2022; Eriksson, 2007) was chosen as theoretical perspective for this study. In caritative caring, the focus is on health resources and the patient, and his or her world is placed in the center. According to Eriksson (1987), the purpose of caring is to alleviate suffering and promote health and well-being. Furthermore, the caring relationship between caregiver and patient is central, and caring becomes visible in its absence. In this study, the caring encounter implies openness and sensitivity toward the new family’s lifeworld, and in line with the caritative caring theory, caring is seen as a natural human behavior developed also into professional care (Bergbom, Nåden, & Nyström, 2022; Bondas, 2005). Ontologically, the human being is viewed as an integrated entity that unites body, mind, and spirit, and when the human being has problems, needs, and desires, he or she must be considered in accordance with this (Arman et al., 2015). Caring is constituted of ethos, which reflects basic values such as caritas, inviolability, human dignity, and the holiness of life (Eriksson, 2018). Ethos and ethics are closely related, and when nurses incorporate ethos into their clinical work, caring becomes evident when responsibility for the patients’ dignity is taken (Bergbom, Nyström, & Nåden, 2022). Ethical responsibility is always fundamental, and nurses’ feelings of responsibility in the encounter with the new family might emerge in a variety of ways (Clancy & Svensson, 2007).

Previous research shows that there is a discrepancy between new mothers’ and fathers’ perceived need for care and support, and what they experience receiving (Høgmo et al., 2021; Sacks et al., 2022). It has also been found that fathers’ need for follow-up is not recognized in the same way as the mothers (Finlayson et al., 2023; Høgmo et al., 2023). This is reflected in the research, which largely focuses on mothers’ experiences with postnatal health care (Finlayson et al., 2020; Slomian et al., 2017; Walker et al., 2019). Studies on fathers’ experiences and knowledge about the topic from the nurses’ point of view exist but seems to be more scattered. To contribute to the further development of the knowledge base and the municipal postnatal health care, we find it important to emphasize nurses’ knowledge and perspectives, as they are the ones offering care and support to the families. According to Bondas and Hall (2007), metasynthesis can complement existing empirical studies by offering a new and more complete interpretation of the findings. After an extensive search, and to the best of our knowledge, no previous metasynthesis has focused on nurses’ perspectives on municipal postnatal health care.

### Aim and Research Question

The aim of this meta-ethnography was to integrate and synthesize qualitative studies that illuminate and describe nurses’ perspectives on municipal postnatal health care in high-income countries. The goal is to expand the knowledge base from the nurses’ perspectives and strengthen research-based postnatal care to promote health and wellbeing for the new family, hence the following research question: “What are nurses’ perspectives on municipal postnatal health care in high-income countries?”

## Methods

Meta-ethnography, as described by Noblit and Hare (1988), is used to synthesize qualitative research findings. In order to identify and develop new overarching concepts, models and theories, meta-ethnography systematically compares conceptual data by using an analytic process of translating the studies into one another toward a synthesis (France et al., 2019; Noblit & Hare, 1988). Meta-ethnography is a complex and distinct qualitative methodology that has the potential to lead to new understandings of health care issues, and high-quality meta-ethnographies can contribute to inform evidence-based policy and practice (France et al., 2019). Meta-ethnography aims to interpret and integrate rather than aggregate findings, moving beyond the original qualitative studies included (Bondas & Hall, 2007). Noblit and Hare’s (1988) seven phases of meta-ethnography guided the synthesis. The eMERGE guidelines developed by France et al. (2019) were also adopted to improve the clarity and completeness of meta-ethnographic reporting.

### Data Collection and Analyses

#### Phase I—Getting Started

The current topic is part of a larger research project on parents’ expectations and experiences with public health nursing and child and family health center (CFHC) services. Ethics approval was not required for this meta-ethnography.

When reviewing the literature, it became evident that nurses’ perspectives on postnatal health care have, to some degree, been addressed in the literature, but they need more attention (Alexandrou et al., 2018; Ashford et al., 2017; Slomian et al., 2017). As we focused on nurses’ own perspectives as described in previous qualitative studies, we decided that the most appropriate approach was an interpretative meta-ethnography because it seeks to go beyond the findings of any one study using idiomatic rather than iterative translations to allow for new understandings (France et al., 2019; Noblit & Hare, 1988).

The members of the research team have different clinical, personal, and international experiences which led to fruitful discussions throughout the study. In addition, two of the authors have extensive and varied experiences in qualitative methods, which has been important to ensuring a reflective and critical research process (Bondas & Hall, 2007; France et al., 2019; Noblit & Hare, 1988).

#### Phase II—Deciding What is Relevant

In this phase we developed the inclusion and exclusion criteria. The process of determining the criteria involved discussing relevant studies among the research team, as well as keywords and the preliminary criteria with an academic librarian. We furthermore examined the criteria in light of the research aim and question. We defined municipal postnatal healthcare to include health education and promotion, risk identification and prevention of complications and support for new families. According to the meta-ethnography methodology we have used a purposeful sampling strategy where studies from low- and middle-income countries have been excluded as the context and postnatal healthcare services differ from those of high-income countries. As shown in a qualitative evidence synthesis on factors that influence uptake of routine postnatal care by Sacks et al. (2022), different prioritized topics are studied in different regions of the world and it is possible that certain areas, as well as certain topics in each region, are understudied. While a desire for more psychosocial and emotional support was highlighted in studies from high-income countries, more emphasis was placed on poor physical infrastructure and insufficient resources in low- and middle-income countries (Sacks et al., 2022). The inclusion criteria used in the study selection can be seen in Table 1.

**Table 1.** The Inclusion/Exclusion Criteria.

Inclusion	Exclusion
• Healthcare professionals such as public health nurses, midwives, nurses, or health visitors	• Focus on parents or other family members experiences or perspectives without health care professionals’ perspectives
• Qualitative studies from a municipal postnatal health care context focusing on the health care professionals’ perspectives of the service delivered	• Gray literature, theoretical papers, reviews, editorials, book-chapters, or comments
	• Quantitative and mixed-method studies
• Studies with different perspectives (mothers, fathers, parents, health professionals) when possible, to separate the perspectives of health professionals	• Studies in other languages than English or Scandinavian
	• Studies from low and middle-income countries
• Studies from high-income countries	• Prevention of family violence and child abuse
• Peer-reviewed original qualitative research studies	
• No time limitations	
• English and Scandinavian languages	

**Figure 1.**
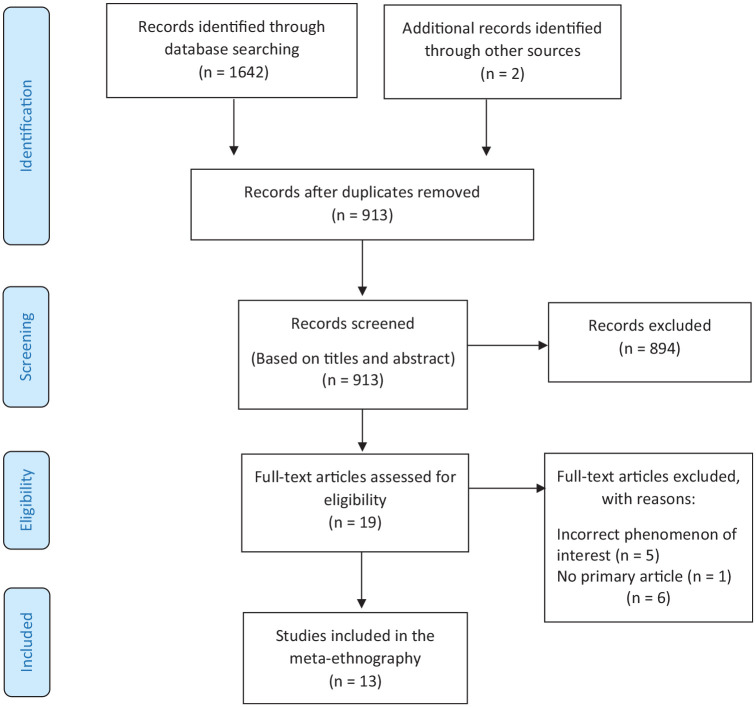
An adapted PRISMA flow-chart of the literature search (Page et al., 2021).

An extensive and thorough literature-search strategy based on the inclusion and exclusion criteria was developed by the first author (BKH) and an academic librarian (KH). No time limitations were set in the search process to gain a broad picture of previous research on the topic and ensure the inclusion of all good quality articles. The same academic librarian performed the search in June 2022, and an updated search in November 2022 (The complete database search strategies are provided in Supplemental File 1). Six electronic databases—CINAHL, Medline, Embase, PsycInfo, Web of Science, and the British Nursing Index—were searched. Reference, citation, and journal searches (Sandelowski & Barroso, 2007) were completed by the first and third author (BKH and TEB). The first author screened the titles and abstracts, and all authors were involved in screening the full texts. Those original qualitative articles considered suitable, according to the research objective, were included. The systematic search and other sources yielded 1644 records. We used the PRISMA flowchart (Page et al., 2021) to describe the process of selection (Figure 1).

#### Phase III—Reading the Included Studies

The analyses were initiated by a repeated reading of the included studies, data extraction and taking note of interpretive metaphors. Quotations from study participants, alongside key concepts or interpretations made by the primary authors, were extracted into a table by the first author. The completed data extraction forms were discussed among the research team. An overview of the included studies and its characteristics is presented in Table 2.

**Table 2.** Characteristics of Included Studies.

First author	Country	Design	Aim/objective	Sample	Participants	Results
Giltenane et al. (2021, 2022)	Ireland	Focus groups	To explore the views and experiences of work environment, quality- and measurement of care provided during a first postnatal visit from the perspectives of mothers and public health nurses.	*n* *=* 24	PHNs Mothers	PHNs perceived as very important for providing support during the first postnatal visit. Relationship building, empowerment and health promotion identified as pivotal to the PHNs’ role, in addition to offer care and advice around physical and psychological wellbeing for mother and infant. Challenges of measuring the quality of PHN practice and challenges of providing a quality service is evident. The PHNs strive to provide a quality service, however the work environment and pressures on PHNs are hindering them from doing so.
				19		
				5		
Aston et al. (2015, 2016)	Canada	Semi-structured interviews	To explore how home visiting programs were organized, delivered and experienced through the everyday practices of PHNs, mothers and managers in Nova Scotia, Canada	*n* *=* 36	PHNs	Relationships seen by the participants as an essential part of supporting mothers and families. The “How-To” of relationship development was discussed. It included being approachable and friendly, and that the approach was strenght-based, client led and respectful. The participants emphasized the importance of focusing on being and feeling“normal,” “confident,” and “non-judged.” Feeling normal and gaining confidence are helping mothers to effectively take care of themselves and their babies.
				16	PH-managers	
				4	Mothers	
				16		
Stewart-Moore et al. (2012)	Northern Ireland & Republic of Ireland	Focus groups & individual interviews	To explore whether professional home post-natal care in NI and ROI meets mothers’ expectations and needs	*n* *=* 58	CM	Mothers’ experiences of postnatal visits, advice given, out-of-hours help, and continuity of care emerged as main themes. A tension between too many or too few home visits suggest that visiting health professionals need to negotiate the visiting patterns with mothers to meet their individual needs.
				12	LS	
					SM	
				5	NI-mothers	
				20		
				20	ROI-mothers	
Kokab et al. (2022)	UK	Focus groups	To explore views and experiences of community midwives delivering post- natal care	*n* *=* 47	CM	Conditions on the postnatal ward and women’s experiences of care in the hospital were factors influencing timing of discharge which resulted in CM managing women and babies with more complex needs. To manage the increased workloads, there was a growing use of flexible approaches to provide care, such as consultations by telephone and postnatal clinics.
				34	CMTL	
				13		
Jansson et al. (2001)	Sweden	Focus groups	To discover how nurses’ views of the first meeting with parents and their new-born child	*n* = 21	PHNs	Three categories were established: Creating trust, creating a supportive climate, and creating a picture of the family’s life situation. The home visit was seen as an important way to establish trust and to obtain a picture of the family’s life situation, which was in turn essential for creating a supportive climate.
				17	CN	
				8		
Engström et al. (2023)	Sweden	Focus groups	To explore professional’s experiences of supporting two mother families in antenatal and child health care	*n* *=* 13	Midwives	One main category was identified: Striving to be open- minded in supporting same sex mothers. Health care professionals described meeting well-prepared mothers, with an equal commitment between each other, and mothers on guard against heteronormative views. Professionals provided support through empowerment by creating a safe environment and aiming at providing equal support or tailored support to same-sex mothers. Professionals expressed that knowledge acquired through education, experience and personal interest all contributed to their competence.
				8	Nurses	
				5		
Homanen (2017)	Finland	Ethnography	To explore practices of parental support in the maternety health care provided by the welfare state	Videotapes/observations of MHC appointments (69), professional team meetings (11), training sessions for HCP (5), family counseling classes for parents-to-be (8), additional documentary material, complementary inter-views with PHNs	PHNs	Parental competence is achieved largely through the “natural” process of experiencing pregnant life. Care practices can be seen as enabling parenthood through respect for this process. Clinics encourage parents-to-be to self-reflect and be self- reliant. Emphasis on self-reflection and self-reliance has previously been interpreted as the state adoption of therapy culture, and as a response to market demands for the welfare state to offer to and require of its citizens more autonomy and choice. The parental subject emerging from the practices of this welfare service cannot be reduced to a neoliberal reflexive individual for whom parenthood is an individual project and who is to blame for individual shortcomings, or being pushed to conform to an idealized parent figure derived from collective ideas of good parenthood.
Eikemo et al. (2023)	Sweden	Individual semi- structured interviews, survey with open ended questions	To describe new mothers’ and midwives’ experiences and perceptions of a new coordinated postnatal care intervention in a midwifery clinic	*n* *=* 192	Midwives Women	The experiences of the new postnatal care model were interpreted by means of management, informational and relational continuity. There is an overarching need for more focus how pregnant women are given the opportunity to prepare for the time after childbirth and how their postpartum well-being and health could be improved by midwifery care. Using a structured and coordinated care model as a midwife that includes planning for the postnatal period together with the pregnant woman at the end of pregnancy may be a good and relatively easy way to create continuity and thus ensure satisfaction and confidence in expectant and new mothers.
				9		
				183		
Barimani and Hylander (2012)	Sweden	Interviews, participant observations and documents	To investigate strategies for continuity of care for expectant and new mothers as experienced by midviwes/CHC nurses and mothers, and elaborate on the preliminary substantive grounded theory model of “linkage in the chain of care.”	*n* *=* 4	Midwives, CHC nurses, and mothers	The main concern of the interviewed CHC nurses and midwives was to ensure that mothers benefited and experienced support and continuity, which was also consistent with mothers’ expectations. However, some nurses and midwives only had visions of strategies for continuity of care, whereas others actually implemented strategies for continuity. When strategies for continuity of care were implemented, the CHC nurses and midwives acted together in joint activities through several different joint practices guided by the three strategies for continuity, transfer, adjustment, and establishing and maintaining a relation.
				1		
				9		
				11		
				21		
Rollans et al. (2016)	Australia	Observations, fieldnotes, interviews, and discussion groups	Explore how midviews and CFHNs interact with the partners or others who attend appointments with pregnant or new mothers when psychosocial assessment or depression screening will be conducted, describe women’s perceptions of their partners’ experience of this practice.	*n* *=* 65	Midwives, student mid-wives, CFHNs, and women	Four key themes were revealed; “negotiating partner exclusion,” “partial inclusion,” ‘women’s business or a couple concern?’ and “they know anyway.” Partner involvement appeared to be challenged particularly by mandatory inter-personal violence screening, which according to health service policy, is to be conducted confidentially. Partner inclusion was minimal. Clinicians’ personal attributes and clinical skills played a significant role in whether partners were successfully included. Various practical issues also impeded partner inclusion, most notably the requirement to assess sensitive issues such as IPV and obstetric history with the woman confidentially. This study highlights the need for the development of site-specific policies and procedures to facilitate the inclusion of partners in perinatal psychosocial assessment and depression-screening appointments.
Levorstad et al. (2022)	Norway	Focus groups	To explore midviews’ experiences of an organizational change in early postpartum care services from hospital to home-based care in Norway.	*n* *=* 10	Midviews	The following three main themes was identified: (1) Unclear leadership, (2) Increased continuity of care, and professional growth, and (3) The midwives being solution oriented. The continuity in care allowed the midwives to thrive professionally and obtain closure after following the women through their pregnancies and increasing their knowledge of breastfeeding and postpartum care. Home visits were seen as a great advantage and important to focus on the birth experience while not losing sight of the big picture of becoming a new family. The midwives saw limited time resources as a challenge in relation to home-based postpartum care. The midwives suggested a further strengthening of the home-based postpartum care service.

*Note.* CM = community midwives; LS = lead supervisors; SM = service manager; CMTL = community midwife team leader; CN = child nurse; MHC = mother health center; HCP = health care personnel; CHC = child health center; CFHN = child and family health nurses.

#### Phase IV—Determining How the Studies are Related

After several readings, we determined how the studies were related by juxtaposing the major findings (Noblit & Hare, 1988). The authors made an initial assumption about the relationship between the included studies being analogous and performed the data extraction by using line-by-line coding (France et al., 2019).

#### Phase V—Translating the Studies Into One Another

By translating the studies into one another, we compared the findings from one study with those from another and themes were clustered and reflected upon. This process was not linear as we went back and forth between the primary studies and the findings, comparing similar findings between the studies (Noblit & Hare, 1988). The translation process is exemplified in Table 4 (see Supplemental File for the translation process), starting with the index paper assessed as having rich findings (France et al., 2019).

#### Phase VI—Synthesizing Translations

Synthesizing in meta-ethnography refers to making a whole into something more than the parts alone imply (Noblit & Hare, 1988), and when analyzing the translations, we moved beyond the findings of the individual studies to a second level of synthesis (Bondas & Hall, 2007; France et al., 2019). The interpretive process has the potential to create metaphors for a deeper understanding, and by translating each primary study into each other, three themes were developed, as shown in Table 5 (see Supplemental File for the translation to sub-themes, themes, and overarching metaphor). The synthesis provides a new and in-depth understanding of nurse’s experiences and perspectives concerning municipal postnatal health care in high-income countries.

#### Phase VII—Expressing the Synthesis

After analogous and refutational translations, a new interpretation encompassed all the translations into an overarching metaphor *Being a “warrior” to care for the new family.* This meta-ethnography is not written solely for an academic audience. To effectively communicate the synthesis to both professionals, lay caregivers, families, and politicians, the language and concepts are used to enable the readers to see the phenomena in terms of others’ interpretations and perspectives (Noblit & Hare, 1988).

## Results

Based on the findings in the 13 included studies (Table 2), the overarching metaphor *Being a “warrior” to care for the new family* was adopted. The metaphor is inspired by the yoga pose “the warrior” picturing how the nurses in the included studies described a stretching and pulling of boundaries related to providing best possible care for the new families. The overarching metaphor was identified and validated in the translation process, where the caregivers internal and external possibilities for caring was perceived as challenged and stretched. By choosing this metaphor, we want to express that stretching boundaries can offer unique opportunities and contribute to growth and health promotion. At the same time, it might contribute to various challenges which, on the other hand, can weaken the quality of care and, for the nurses, represent an increased risk of being “overstretched.” The overarching metaphor *Being a “warrior” to care for the new family* is accompanied by three main themes: *Stretching human boundaries, Stretching system boundaries*, and *Stretching knowledge boundaries.*

### Stretching Human Boundaries

The first theme, *Stretching human boundaries*, reflects how the nurses described the first encounter with the parents and how the home setting contributed to facilitating a mutual encounter where boundaries between the care receiver and the professional caregiver were stretched. By stretching their human boundaries, the nurses were able to connect and communicate with the family in a trustful and supportive way. This theme constitutes two subthemes: *Being caring and authentic* and *Creating a picture of the family’s situation during the home visit.*

#### Being Caring and Authentic

The nurses perceived themselves as guests in the new families’ homes and described encountering parents as more relaxed in their own home environment (Aston et al., 2015; Giltenane et al., 2021, 2022; Jansson et al., 2001). The first home visit facilitated communication, which was seen as very important in creating a reciprocal relationship and as a necessity for establishing trust and support (Aston et al., 2015; Giltenane et al., 2021, 2022; Jansson et al., 2001). Stretching toward each other as equal human beings was perceived by the nurses as an important act in creating a mutual relationship. The following quote from a focus group discussion highlight some of the participants’ views on the critical part of the first meeting with parents and their newborn in their home: “I’ve never thought of it as bonding. But you’re right in saying that a relationship should feel reciprocal. We also bond from our direction” (Jansson et al., 2001, p. 144). By being authentic and friendly, the nurses created a supportive climate characterized by confirmation through listening and identifying individual needs and family resources, and they experienced that a good relationship influenced the efficacy and quality of care (Aston et al., 2015; Jansson et al., 2001). Establishing relationships with families from different cultures was described as both positive and difficult where language barriers and a lack of translators threatened the relationship and quality of care (Giltenane et al., 2022).

At the same time, it was also acknowledged that the home environment could hinder confidentiality, security, and quality of service delivered: “(. . .) everybody stays in the room, and you are sort of saying “is there anywhere maybe we could go where I could examine the baby?” “No, no it is fine here” and you try and offer (. . .) (Giltenane et al., 2022, p. 208). The nurses described the lack of privacy when examining the mother’s breasts or perineum as the main reason for challenging quality.

A movement between closeness and distance between the parents and the nurse was evident in the included studies (Aston et al., 2015, 2016; Giltenane et al., 2021, 2022; Homanen, 2017; Jansson et al., 2001; Kokab et al., 2022; Stewart-Moore et al., 2012). By describing themselves as humble and reserved when visiting the families in peace and privacy, the nurses draw a picture of a genuine encounter between people on equal footing, which enabled a safe climate important for unraveling any concerns (Giltenane et al., 2021, 2022; Jansson et al., 2001). “They’re on their home ground, they have the advantage of being at home. You take off your shoes in the hall, we come to them in our stocking soles, we are guests, and I think that’s the key” (Jansson et al., 2001, p. 145). The nurses focused on strength-based interactions with a flexible and client-led discourse where the mother was seen as the expert. A shift in the power dynamic encouraged mothers to feel they had more “control” and autonomy, and nurses described recognizing new mothers’ perception of them as an authority person who might judge them: “I am not going in there as an authority figure [. . .] and so it makes them feel more comfortable [. . .] so if I want to come back, they’re more willing to have me back” (Aston et al., 2015, p. 20). Although the nurses perceived themselves and the parents as equal partners, it was seen as important to keep some distance and allow parents to trust their own resources (Aston et al., 2015; Homanen, 2017).

#### Creating a Picture of the Family's Situation During the Home Visit

The home visit was deemed invaluable because it gave a multidimensional picture of the family’s life situation. New mothers need to know and be reassured; they want to do the “right things” and be normal, and by listening and giving support, the nurses empowered mothers to care for themselves and the baby (Aston et al., 2016; Giltenane et al., 2021; Stewart-Moore et al., 2012). The nurses strived to be good listeners and signaled to the new parents that they stretched themselves toward understanding the parents’ situation and needs: “I think you can relieve a lot of anxiety. Often parents have lots of questions and it is just to give them an opportunity to ask those” (Giltenane et al., 2021, p. 9). The home visit also enabled families to talk about the birth experiences, while at the same time being aware of not losing sight of “the big picture of becoming a new family” (Levorstad et al., 2022). It was also described as important to emphasize the naturalness of living with small children and support the parents in their faith in themselves as many parents feel insecure in their new roles as parents and grandparents are no longer as accessible as before (Giltenane et al., 2021; Jansson et al., 2001). Some nurses described having practical and intuitive ways of addressing the new life with a baby and parental identity. They saw this transition as parental psychological and emotional choices and supported the process of coming to know parenthood by using psychological knowledge to approach parenting, but they hesitated to provide content to desirable family life (Homanen, 2017). Self-reliance and parenthood were perceived as an individual, intuitive, and natural processes. Moreover, there was a conviction that there are many ways of doing parenthood (Homanen, 2017).

### Stretching System Boundaries

The second theme, *Stretching system boundaries*, represents how the surrounding system affected the delivery of postnatal health care and how different system boundaries were pulled and stretched. The theme is accentuated by three subthemes: *Minimizing home visits as a solution in managing increased workload, Inviting parents’ freedom of choice and Continuity in listening to the unfolding story.*

#### Minimizing Home Visits as a Solution in Managing Increased Workload

Even though home visits are described by parents and health professionals (Aston et al., 2015; Giltenane et al., 2021, 2022; Jansson et al., 2001) as a positive experience that promotes safety, trust and relationship building, this study also shows how nurses stretch system boundaries to manage the increased workload by working beyond their scheduled working hours and changing or minimizing the postnatal health care offers. Early discharge and a tight timeframe increased the postnatal care workload, which again affected the quality of care and service provided, and limited municipal resources affected both planning and care (Giltenane et al., 2022; Kokab et al., 2022; Levorstad et al., 2022). “Too early” and problematic discharges were evident, and the nurses felt there was nothing they could do about the situation:

“If a lady’s had a really traumatic time and then she’s sent home quite early, I really worry about those women, about what’s going to happen, what support have they got at home? Have they got adequate support from mum, partner, you know, is the partner on paternity leave, is he going to be there for her? Or is she going to go home on her own and be left with this baby to cope and then end up really depressed?” (Kokab et al., 2022, p. 4).

Minimizing home visits, postnatal clinics and phone calls were described as a practical solution in managing increased workload. At the same time, it was perceived that to limit parental and family stress in the early postnatal period, a further strengthening of home-based postnatal care was needed (Levorstad et al., 2022). Despite a heavy workload, the nurses were confident in the care provided during the first postnatal visit, and this visit was prioritized even if it resulted in unpaid working hours to provide quality service (Giltenane et al., 2022; Kokab et al., 2022).

#### Inviting Parents’ Freedom of Choice

Although some nurses in the included studies emphasized parents’ right to choose the home or clinic for the first meeting, they described a practice where they hesitated or failed to inform the parents about their right to choose based on their experiences and knowledge of all the advantages associated with the home visit. The importance of parents being able to choose the home or clinic for the first meeting was highlighted in one of the studies, but a discrepancy between nurses expressed attitudes and practice was evident (Jansson et al., 2001). Some nurses hesitated to inform parents about their right to decide the form of visit or did not ask or inform them about the purpose of the home visit. In one of the focus groups, it was stated that: “It is terribly important that the first meeting should be a home visit. Yes, the home visit, the information, and the contact I get there are worth their weight in gold” (Jansson et al., 2001, p. 148), while another nurse described a practice where she in reality did not give the parents the opportunity to choose: “I haven’t asked. Nobody has protested” (Jansson et al., 2001, p. 148).

Postnatal clinics were seen as important for support groups and network building. A perceived risk for non-holistic care, which includes the lack of opportunity to identify safeguarding concerns in a clinic instead of in the home environment, was described (Kokab et al., 2022). Even though they understood the need for postnatal clinics, nurses reported concerns when new mothers did not attend the postnatal clinics for early visits.

#### Continuity in Listening to the Unfolding Story

Listening was described as a quality of care, and the nurses strived to be good listeners as they stretched themselves toward the parents to identify their experiences, needs, and wishes. In the present study, continuity of care was highlighted as a great advantage for both families and nurses, and when acting together as a team, it was easier for the nurses to ensure necessary adjustments and the transfer of information. A general difficulty in verbalizing the required competence was evident, and this made acquired practical competence difficult to measure and describe (Giltenane et al., 2022; Jansson et al., 2001). According to the nurses, listening, supporting, and continuity of care were perceived as a quality of postnatal care. For many people part of the competence is that there is continuity because you don’t ask all your questions the first time. “When you feel trust in the nurse, the other questions will come” (Jansson et al., 2001, p. 147). Joint action between nurses and midwives facilitated a chain of care perspectives, and professionals as well as the parents benefited from this continuity (Barimani & Hylander, 2012). Midwives valued being able to follow the women after birth, and mothers with a special need for support could be introduced to the child health center (CHC) before birth (Barimani & Hylander, 2012; Eikemo et al., 2023; Levorstad et al., 2022). For some of the midwives, the continuity of care represented increased knowledge and made them thrive professionally in addition to obtain closure after following the women through their pregnancies (Levorstad et al., 2022). Acting together as a team enabled the nurses and midwives to transfer information and make necessary adjustments. “The best thing is that we are under the same roof. I can walk into the room of the midwife if I haven’t got all the information I need. I can knock on the door right away, which makes it easier. They bring in mothers to us as well, so they know who they are going to meet afterward.” (Barimani & Hylander, 2012, pp. 6–7).

### Stretching Knowledge Boundaries

The third theme, *Stretching knowledge boundaries*, underlines the nurse’s role in supporting the new parents’ process of coming to know parenthood within the frames of a relationship based on equality and parents’ autonomy while simultaneously managing risks and establishing security through prevention and health promotion. The theme is elaborated by two subthemes: *Seeing mother as the expert and father as a bystander* and *Striving to be open-minded and inclusive.*

#### Seeing Mother as the Expert and Father as a Bystander

In most of the included studies, the focus was on the mother and baby (Aston et al., 2015, 2016; Barimani & Hylander, 2012; Eikemo et al., 2022; Giltenane et al., 2021, 2022; Stewart-Moore et al., 2012). The mother was seen as the expert, and parental identities were achieved through a supported process of coming to know (Aston et al., 2015, 2016). While women acquired parental identity by experiencing pregnancy, partners (implicitly men) needed an invitation to start this process (i.e., ultrasound) (Homanen, 2017). Although self-reliance and parenthood were perceived as a natural process, the nurses described that mothers experienced considerable pressure to do things right and determine what is “normal” (Aston et al., 2016; Giltenane et al., 2021). What constituted “normality” was determined by average standards where an average family is one within a normal distribution on risk scales. Thus, autonomy and privacy were perceived as individual rights of the family as long as they were within the frame of “normality” (Homanen, 2017). Reflecting on their parental journey was described as sufficient to provide some parental competence, while its absence was considered a risk factor in itself (Homanen, 2017).

Support was seen as crucial for some mothers’ ability to continue breastfeeding (Aston et al., 2016; Engström et al., 2023). By supporting mothers’ feeding choices, nurses were cautious about how they could help them avoid feeling guilty when they chose not to breastfeed. “She’s [the mother] making a conscious decision not to breastfeed and I’m going to support her during that. And she’s just as important” (Aston et al., 2016, p. 7). The nurses acknowledged and empowered mothers by providing information, education and practical hands-on-help, something that gave the mothers self-confidence in their desire to know that they were doing a good job (Aston et al., 2016; Giltenane et al., 2022). The non-judgmental approach helped the mothers feel confident in their choices and more comfortable in the support offered. Although the nurses supported the process of coming to know parenthood and used more or less standardized ways of supporting transitions and screening for problems, it was evident that partners were often excluded, while focus was on the mother and baby (Homanen, 2017; Rollans et al., 2016).

#### Striving to be Open-Minded and Inclusive

The nurses strived to be open-minded, being aware of the terminology used, and they also tried to create an equal and inclusive environment for all parents (Eikemo et al., 2022; Giltenane et al., 2021; Rollans et al., 2016). Concurrently, it was perceived as a balancing act to acknowledge and strengthen same-sex parents in their different parental roles (Engström et al., 2023). Same sex-mothers were often experienced as well-read and knowledgeable with a plan for shared commitment and parenting. At the same time, nurses described parents who unknowingly felt they had to defend their parenthood: “I know that LGBTQ people must constantly defend themselves, because you’re constantly questioned” (Engström et al., 2023, p. 5). In one of the studies, nurses expressed a strong interest in LGBTQ issues and stated that they needed more knowledge, and that the topic was lacking in both nursing and midwifery education (Engström et al., 2023).

Postnatal depression was perceived as an important issue to discuss with mothers, and the nurses described it as their responsibility to prevent and detect postnatal depression and contribute to reducing mothers’ level of stress (Aston et al., 2016; Giltenane et al., 2021; Rollans et al., 2016). There were different ways of approaching male partners when carrying out psychosocial assessments and screening for PND, and it often had a negative impact on both parents when the partner was asked to leave the room. “There’s a whole load of questions, about your physical health and there’s a section on your emotions and social supports and we’ll ask you (looks to partner) to leave the room during that section” (Rollans et al., 2016, p. 306). The partner was not excluded during the first home visit, but assessment and screening practices were adapted if the partner or others were present. The nurses’ experiences and knowledge affected the different strategies used to minimize the impact on the partner or other excluded persons and promote partial inclusion (Engström et al., 2023; Rollans et al., 2016). When present, the partner interacted and wanted to be involved and provide support. At the same time, the presence of a partner in these interviews could also be negative and hinder the disclosure of intimate partner violence (Rollans et al., 2016). Clearer policies and procedures to facilitate the inclusion of both parents in perinatal psychosocial assessment and depression-screening were needed. The rationale for excluding partners was perceived as unclear or irrelevant because partner may “know anyway” what is being discussed (Rollans et al., 2016).

## Discussion

The metaphor *Being a “warrior” to care for the new family* is the overarching theme for this discussion. The metaphor is accompanied by three main themes, stretching human, system, and knowledge boundaries, contributing to broadening the picture of the nurses’ perspectives of municipal postnatal health care. The result of this meta-ethnography describes how the frames nurses in high-income countries work within might affect their internal and external possibilities for maneuver in both positive and negative ways (e.g., through being caring and authentic when home visiting and by minimizing home visits as a solution in managing increased workload).

When boundaries between the health care professional and the care receiver are stretched, the nurses experience that a common ground for establishing a reciprocal relationship is created. The home environment acts as an important prerequisite in facilitating communication and connection in the first meeting with the parents. Perceiving themselves as guests in the families’ homes and meeting more relaxed parents on their home ground contributed to a safe climate where health professionals and parents could get to know each other on an equal footing. When creating a mutual relationship, stretching toward each other as human beings was perceived by nurses as an important act. Eriksson (2018) states that caring is sharing, and true sharing is seen as an act of mercy. Sharing means togetherness and something giving energy to growth, development, and inner strength. At the same time, it is important to remember that the interactions between nurses and patients are asymmetrical (Delmar, 2012). The nurse has a role in the relationship with the patient that differs from that found between two friends whose connection is fully voluntary. This is because their interactions are based on a dependence and power relation (Eriksson, 2018).

When returning home from hospital with a newborn child, parents describe an increased need for information, care, and support (Hrybanova et al., 2019; Sacks et al., 2022). Being treated with care and respect by familiar and trusted health care professionals is emphasized as particularly important during the postnatal journey. Our study illuminates that the home visit was perceived by nurses as invaluable in giving a greater and multidimensional picture of the family’s lifeworld (Aston et al., 2016; Giltenane et al., 2021; Stewart-Moore et al., 2012), and in line with the caritative caring theory, the goal of the nurses was to promote health and alleviate suffering through a caring communion with the new family in the postnatal period (Bondas, 2005). At the same time, there was a predominant focus on mother and child in most of the included studies, which may indicate that there is a mismatch between how the nurses wanted to meet the new families and how they were actually encountered.

Listening was described as a quality of care, and the nurses strived to be good listeners as they stretched themselves toward the parents to identify their experiences, needs and wishes. In the present study, continuity of care was highlighted as a great advantage for both families and nurses, and when acting together as a team, it was easier for the nurses to ensure necessary adjustments and the transfer of information. A study on maternal needs following childbirth from both parents’ and professionals’ perspectives (Slomian et al., 2017), revealed a discrepancy between how professionals and parents perceived new mothers’ information and care needs. Having different perspectives on important issues such as informational, practical, and emotional needs, might create a gap between the caregiver and the care-receiver in the postnatal period. Balancing closeness and distance are important aspects, and as shown in the results of this meta-ethnography (Aston et al., 2015; Homanen, 2017), focus must be placed on establishing a close caring relationship where care needs and concerns can be unraveled, while simultaneously keeping some distance, allowing parents to develop self-reliance and trust in their own resources (Delmar, 2012). The included studies revealed that *stretching human boundaries* contributes to unique opportunities for good postnatal care, and this has the potential to offer new insight for parents as well as health professionals.

At the same time, the independent nature of public health nursing and municipal postnatal health care can contribute to health professionals’ feelings of responsibility. As defining boundaries for involvement is not always an easy task (Clancy & Svensson, 2007), it is important for the nurses to be aware of such boundaries of involvement, as one aspect of ethical responsibility involves the ability to “say no” to other people’s demands. This study’s results show that quality of care and service provided by the nurses was affected by their postnatal care workload. Early discharge from hospital has become common in many western countries (Campbell et al., 2016; Jones et al., 2021), contributing to an increased need for qualified health care personnel in the community. A tight timeframe and limited municipal resources were evident in several of the included studies, and this, together with some experiences of problematic and “too early” discharges were factors affecting the planning and quality of nursing care. The first days and weeks after birth are crucial for building relationships and establishing behaviors that affect long-term infant development and health. A globally recognized goal is that all new parents, their babies and families should have, as a significant end point, a “positive postnatal experience” (World Health Organization, 2022 World Health Organization (2022). However, this goal might be difficult to reach, as many nurses are in danger of being overstretched due to the introduction of New Public Management with increased demands for financial efficiency, standardization, and reporting (Selberg, 2013). The results show that for nurses working in the municipal postnatal healthcare in high-income countries, there is a fine balance between stretching in a way that contributes to health and development and the risk of stretching too far.

Some of the nurses described home visits as especially important in reducing parental stress in the early postnatal period and even suggested a further strengthening of home-based care. At the same time, a solution in managing tight timeframes and heavy workload was reducing home visits and instead offering phone calls and consultations at postnatal clinics. The nurses perceived the clinics as important in network building and for support groups but also described a concern for those mothers, who did not attend the postnatal clinics for early visits. When unable to visit the new family in their own home environment, the nurses experienced a risk of not gaining a comprehensive picture of the family and their situation.

The caring relationship was central in the included studies, and when listening to the new mothers and fathers’ experiences and needs, the nurses were able to alleviate suffering and promote health through a caring communion (Bondas, 2005). On the other hand, when stretching system boundaries and not being able to offer the best possible care, the nurses experienced compromising their professional and ethical values, which again threatened the quality and capacity of care.

Most of the included studies had their focus on the mother and baby and there are paradoxical findings in the studies that show the difference between the nurses’ wished-for-ideals to encounter and care for the whole family, and the reality of a busy everyday situation that leaves little space for change and reflection. At the same time, our study shows that nurses are stretching toward a more inclusive postnatal health care. Mothers were seen as the expert and acquired parental identity by experiencing pregnancy, while partners (implicitly men) had to be “invited in” to start this process (Aston et al., 2015, 2016; Homanen et al., 2017). Although the nurses supported the process of coming to know parenthood and tried to be open-minded and create an inclusive and equal environment, partners often were excluded. This result is in line with other studies showing that the fathers’ needs for support and care are not emphasized to the same extent as mothers during the postnatal period (Høgmo et al., 2021; Solberg et al., 2023).

In one of the studies, the nurses called for more knowledge about LGBTQ families (Engström et al., 2023). It was described as challenging to acknowledge and strengthen same-sex parents in their different roles as new parents, which contributed to the fact that the non-birthing parent was at risk of not receiving the same support and care as the birthing woman. A lack of knowledge might leave parents feeling marginalized and excluded, giving the impression of an unequal and heteronormative health care service (Andersen et al., 2017; Klittmark et al., 2019). The nurses highlighted the importance of psychosocial assessment and PND-screening as important tasks in postnatal care and as found by Cameron et al. (2016), maternal postpartum depression is a significant risk factor for paternal depression. The nurses saw it as an important issue to discuss with the mothers but found it difficult to approach male partners and described that it often affected both parents negatively when the father was asked to leave the room during the consultation. The results revealed that more knowledge, clearer procedures, and policies that include partners in the work with postnatal depression and intimate partner violence were evident and necessary to secure a best possible care for the new family. As described initially, ethos makes us strive to understand the other human being, and in caring it implies practising openness, wakefulness and reverence (Bergbom, Nyström, & Nåden, 2022). From a caring science perspective, this means that it is the nurses’ responsibility to protect the new parents’ dignity and prevent and alleviate suffering so that caring becomes evident. Caritative caring theory underpins the caring power, which is manifested in caring for the other human being based on faith, hope and love, and where theory and practice are united by ethos (Eriksson, 2018).

## Strengths and Limitations

The strengths of this meta-ethnography are based on the rigorous methodology and literature review and the ability to contribute new knowledge by bringing together more information than that found in just a singular study. Following the seven-phase approach developed by Noblit and Hare (1988), and the eMerge reporting guidance (France et al., 2019), contributes to ensuring a more complete and transparent reporting. In this study, the focus has been on going beyond the aggregation of findings to interpret, integrate and create new knowledge (Bondas & Hall, 2007). The included studies were evaluated using the CASP quality appraisal tool (Critical Appraisal Skills Program, 2018), and the search strategy had no time limit, was open to Scandinavian languages in addition to English, and allowed for the inclusion of both PHNs and midwives, which made it possible to include studies from different timepoints and contexts. The team conducting this meta-ethnography included experienced researchers, both on the topic explored and on the methodology. All of the authors contributed to critical and fruitful discussions and the authors’ different background of experience, both contextual and international, played a significant role in the analysis and subsequent synthesis. The themes and the overarching metaphor were analyzed and discussed in repeated meetings led by the first author and in line with Sandelowski and Barroso (2007), think-aloud strategies as well as negotiations were helpful in this phase of the analysis. As described by Beck (2022) metaphors serve the purpose of being a bearer of meanings that can compress rich description into a word or a phrase. Using a metaphor to visualize the interpretation of the nurses as warriors stretching human, system and knowledge boundaries is considered a strength in meta-ethnography and might contribute to a deeper understanding of the phenomenon under study (Noblit & Hare, 1988).

Since the aim of the study was to investigate the municipal public postnatal healthcare services, studies related to early intervention programs for first-time mothers such as Nurse Family Partnership were deliberately excluded. Even though a comprehensive systematic literature search was undertaken, it is possible that not all relevant studies were retrieved, and the inclusion of studies from different countries with different health care systems might affect the transferability of findings. Another potential limitation is that all the included studies were conducted in westernized countries including Canada and Australia. Thus, it can be argued that the included studies in this meta-ethnography only represents the perspectives and expectations of a westernized culture.

## Conclusion

The overarching metaphor *Being a “warrior” to care for the new family* symbolizes how nurses in high-income countries are stretching different boundaries while striving to care for the new family, and their willingness to “go the extra mile” to provide a quality service. This meta-ethnography describes the possibilities and challenges related to nurses’ perceived internal and external possibilities for caring. Human boundaries are stretched toward a caring relationship with the family facilitated by the home environment, which is seen by the nurses as a significant and invaluable prerequisite. System boundaries are continuously stretched due to early discharges, tight timeframes, and heavy workload in municipal postnatal health care, challenging the continuity and quality of care. Being a warrior pushing and stretching system boundaries and balancing between their ideals and reality while striving to care for the new families, puts the nurses at risk of being overstretched. A desire for more knowledge about LGBTQ parents’ transition to parenthood is evident, and clearer policies and procedures related to enabling the inclusion of fathers and partners in psychosocial assessment and postnatal depression screening are needed.

More focus on different cultural traditions, family constellations and the inclusion and care of the father or non-birthing parent is suggested. This requires both qualified and skilled nurses and resourced and flexible health systems recognizing the individual needs of new parents and families. We hope that our results encourage a change in education and clinical practice to ensure a further development of the municipal postnatal health care.

## Supplemental Material

sj-docx-1-gqn-10.1177_23333936231218843 – Supplemental material for Being a “Warrior” to Care for the New Family: A Meta-ethnography of Nurses’ Perspectives on Municipal Postnatal Healthcare Click here for additional data file. Supplemental material, sj-docx-1-gqn-10.1177_23333936231218843 for Being a “Warrior” to Care for the New Family: A Meta-ethnography of Nurses’ Perspectives on Municipal Postnatal Healthcare by Bente Kristin Høgmo, Marit Alstveit and Terese Bondas in Global Qualitative Nursing Research

sj-docx-2-gqn-10.1177_23333936231218843 – Supplemental material for Being a “Warrior” to Care for the New Family: A Meta-ethnography of Nurses’ Perspectives on Municipal Postnatal Healthcare Click here for additional data file. Supplemental material, sj-docx-2-gqn-10.1177_23333936231218843 for Being a “Warrior” to Care for the New Family: A Meta-ethnography of Nurses’ Perspectives on Municipal Postnatal Healthcare by Bente Kristin Høgmo, Marit Alstveit and Terese Bondas in Global Qualitative Nursing Research

sj-docx-3-gqn-10.1177_23333936231218843 – Supplemental material for Being a “Warrior” to Care for the New Family: A Meta-ethnography of Nurses’ Perspectives on Municipal Postnatal Healthcare Click here for additional data file. Supplemental material, sj-docx-3-gqn-10.1177_23333936231218843 for Being a “Warrior” to Care for the New Family: A Meta-ethnography of Nurses’ Perspectives on Municipal Postnatal Healthcare by Bente Kristin Høgmo, Marit Alstveit and Terese Bondas in Global Qualitative Nursing Research

sj-docx-4-gqn-10.1177_23333936231218843 – Supplemental material for Being a “Warrior” to Care for the New Family: A Meta-ethnography of Nurses’ Perspectives on Municipal Postnatal Healthcare Click here for additional data file. Supplemental material, sj-docx-4-gqn-10.1177_23333936231218843 for Being a “Warrior” to Care for the New Family: A Meta-ethnography of Nurses’ Perspectives on Municipal Postnatal Healthcare by Bente Kristin Høgmo, Marit Alstveit and Terese Bondas in Global Qualitative Nursing Research
