# Supplementary material for: Being a “Warrior” to Care for the New Family: A Meta-ethnography of Nurses’ Perspectives on Municipal Postnatal Healthcare
Source: Glob Qual Nurs Res. 2023 Dec 25;10:23333936231218843. doi: 10.1177/23333936231218843 (PMC10750548; doi:10.1177/23333936231218843)
Supplement: sj-docx-1-gqn-10.1177_23333936231218843 – Supplemental material for Being a “Warrior” to Care for the New Family: A Meta-ethnography of Nurses’ Perspectives on Municipal Postnatal Healthcare [file sj-docx-1-gqn-10.1177_23333936231218843.docx]

**Supplementary file**

Table 4. The translation process (examples)

| Jansson et al (2001)  INDEX PAPER | Aston et al (2015) | Aston et al (2016) | Giltenane et al (2022) | Stewart-Moore et al (2012) | Kokab et al (2022) | Giltenane et al (2021) | Engström et al (2022) | Homanen (2017) | Eikemo et al (2022) | Barimani & Hylander (2012) | Rollans et al (2016) | Levorstad et al (2022) | Translation |
| --- | --- | --- | --- | --- | --- | --- | --- | --- | --- | --- | --- | --- | --- |
| 1.  Home visit important to create trust, establish a supportive climate and gain an idea of the family's life situation | 1.  Creating positive relationships during the postpartum home visit | **-** | 1.  First postnatal home visit important to unravel any concerns | **-** | **-** | 1.  Building relationship is “key”, mindful of being visitor in family's homes | **-** | **-** | **-** | 1.  ‘‘It was wonderful when the midwife and nurse came home visiting together so I did not have to get to know an entirely new person’’ | 1.  Partners were not excluded by the nurse at the first home visit | 1.  Home-based postnatal care seen as a great advantage for the family | The home visit enables support and relationship building |
| **-** | **-** | **-** | **-** | **-** | **-** | 8.  Sensitive in communicating with parents in order not to jeopardise the relationship | 8.  Creating an inclusive environment, providing equal support to all parents | **-** | **-** | **-** | 8.  Trying to minimize impact of partner being excluded and to promote partial inclusion | **-** | Striving for inclusion and equality in care |
