# Supplementary material for: Being a “Warrior” to Care for the New Family: A Meta-ethnography of Nurses’ Perspectives on Municipal Postnatal Healthcare
Source: Glob Qual Nurs Res. 2023 Dec 25;10:23333936231218843. doi: 10.1177/23333936231218843 (PMC10750548; doi:10.1177/23333936231218843)
Supplement: sj-docx-2-gqn-10.1177_23333936231218843 – Supplemental material for Being a “Warrior” to Care for the New Family: A Meta-ethnography of Nurses’ Perspectives on Municipal Postnatal Healthcare [file sj-docx-2-gqn-10.1177_23333936231218843.docx]

**Supplementary file**

**Table 2.** *Critical appraisal of included studies (CASP).*

Critical appraisal questions

Article 1 2 3 4 5 6 7 8 9 10

| Giltenane et al (2021) | Y | Y | Y | Y | Y | C | Y | Y | Y | Y |
| --- | --- | --- | --- | --- | --- | --- | --- | --- | --- | --- |
| Aston et al (2015) | Y | Y | Y | Y | Y | N | Y | Y | Y | Y |
| Aston et al (2016) | Y | Y | Y | Y | Y | N | Y | Y | Y | Y |
| Giltenane et al (2022) | Y | Y | Y | Y | Y | N | Y | Y | Y | Y |
| Stewart-Moore et al (2012) | Y | Y | Y | Y | Y | N | Y | Y | Y | Y |
| Kokab et al (2022) | Y | Y | Y | Y | Y | N | Y | Y | Y | Y |
| Jansson et al (2001) | Y | Y | Y | Y | Y | N | N | Y | Y | Y |
| Engström et al (2022) | Y | Y | Y | Y | Y | N | Y | Y | Y | Y |
| Homanen (2017) | Y | Y | Y | C | Y | N | Y | Y | Y | Y |
| Eikemo et al (2022) | Y | Y | Y | Y | Y | N | Y | Y | Y | Y |
| Barimani & Hylander (2012) | Y | Y | Y | Y | Y | N | Y | Y | Y | Y |
| Rollans et al (2016) | Y | Y | Y | Y | Y | C | Y | Y | Y | Y |
| Levorstad et al (2022) | Y | Y | Y | Y | Y | Y | Y | Y | Y | Y |

Critical appraisal questions: (1) Was there a clear statement of the aims of the research? (2) Is a qualitative methodology appropriate? (3) Was the research design appropriate to address the aims of the research? (4) Was the recruitment strategy appropriate to the aims of the research? (5) Was the data collected in a way that addressed the research issue? (6) Has the relationship between researcher and participants been adequately considered? (7) Have ethical issues been taken into consideration? (8) Was the data analysis sufficiently rigorous? (9) . Is there a clear statement of findings? (10) How valuable is the research? Y = yes, N = no, C = can't tell.
