# Supplementary material for: Being a “Warrior” to Care for the New Family: A Meta-ethnography of Nurses’ Perspectives on Municipal Postnatal Healthcare
Source: Glob Qual Nurs Res. 2023 Dec 25;10:23333936231218843. doi: 10.1177/23333936231218843 (PMC10750548; doi:10.1177/23333936231218843)
Supplement: sj-docx-3-gqn-10.1177_23333936231218843 – Supplemental material for Being a “Warrior” to Care for the New Family: A Meta-ethnography of Nurses’ Perspectives on Municipal Postnatal Healthcare [file sj-docx-3-gqn-10.1177_23333936231218843.docx]

**Supplementary file**

Table 5. Translations to sub-themes, themes, and overarching metaphor

| Translation | Sub-themes | Themes | Overarching metaphor |
| --- | --- | --- | --- |
| Home visit enables support and relationship building  Humble and reserved, see themselves as guests in the families’ homes  Being authentic/yourself and friendly  A mutual meeting between people equal terms  Shifting the historical power dynamic  Communication important in creating a reciprocal relationship  Enables individualized care and better counselling  Privacy and peace in the home setting  Important to keep some distance  Multidimensional picture of the family's life situation  Listening and identifying individual needs and family resources  Can also reduce confidentiality, security, and quality of service delivered  Support the process of coming to know parenthood  Hesitate to thematize desirable family life  Many ways of doing parenthood  Addressing the new life with a baby and parental identity  Reassurance and support  Empowering mothers to care for themselves and the baby  The naturalness of living with small children | Being caring and authentic  Creating a picture of the family's situation during the home visit | Stretching human boundaries | Being a “warrior” to care for the new family |
| “Too early” and problematic discharges  Tight timeframe increase postnatal care workload affects the quality of care  Limited municipal resources  Need further strengthening of the home-based care  Confident in care provided in the first postnatal visit  Minimizing home visits due to increased workload  Postnatal clinics and phone calls  Risk of non-holistic care at clinic  Important for support groups and network building  Choose the home or clinic for the first meeting  Discrepancy seen between nurses expressed attitude and practice  Home visit a great advantage  Concerns when mothers don’t attend clinic  Difficult to verbalize required competence and to measure the first postnatal visit  and relationship building  Meets the mothers needs by listening and giving support  Nurses and midwives can act together  Increased continuity of care  Allowed to thrive professionally  Joint action facilitates a chain of care perspective | Minimizing home visits as a solution in managing increased workload  Inviting to parents' freedom of choice  Listening is a competence too | Stretching system boundaries |  |
| Mother is seen as the expert  Lack of competence, a need for training  Partners (men) need an invitation to start parental identity process  Standardized ways of supporting transitions and screening for problems  What constitutes ‘normality’  Focus on building up the parents  Continue breastfeeding  Strong interest, but limited training and knowledge in LGBTQ issues  Same sex-mothers often well-read  Heteronormatively communication  Need time to reflect and question their own norms and values  Relationship building with families from different cultures - positive and difficult  Language barriers and lack of translators  Partners excluded from the psychosocial assessment/depression screening  Different ways of approaching male partners  Minimize the negative effect of exclusion  Partner interacts and want to be involved  Striving for inclusion and equality in care  Rationale for excluding partner still unclear or irrelevant  Inclusion of partners are needed, try to create an inclusive environment  Responsibility to prevent and detect PND | Seeing mother as the expert and father as a bystander  Striving to be open-minded | Stretching knowledge boundaries |  |
