# Supplementary material for: Being a “Warrior” to Care for the New Family: A Meta-ethnography of Nurses’ Perspectives on Municipal Postnatal Healthcare
Source: Glob Qual Nurs Res. 2023 Dec 25;10:23333936231218843. doi: 10.1177/23333936231218843 (PMC10750548; doi:10.1177/23333936231218843)
Supplement: sj-docx-4-gqn-10.1177_23333936231218843 – Supplemental material for Being a “Warrior” to Care for the New Family: A Meta-ethnography of Nurses’ Perspectives on Municipal Postnatal Healthcare [file sj-docx-4-gqn-10.1177_23333936231218843.docx]

| **Supplementary file** |  |  |  |  |
| --- | --- | --- | --- | --- |

**Table 1. Search strategy**

| Database/source | **Database - CINAHL with Full Text**  Interface - EBSCOhost Research Databases  Search Screen - Advanced Search  Search modes - Boolean/Phrase |
| --- | --- |
| Date of search | 3 June 2022 |
| Search history or procedure | 1. (MH "Midwives+") OR (MH "Nurses") OR (MH "Nurse Midwives") OR (MH "Advanced Practice Nurses") OR (MH "Nurse Practitioners") OR (MH "Family Nurse Practitioners") OR (MH "OB-GYN Nurse Practitioners") OR (MH "Pediatric Nurse Practitioners") OR (MH "Neonatal Nurse Practitioners") OR (MH "Practical Nurses") OR (MH "Registered Nurses") 2. (MH "Community Health Nursing") OR (MH "Home Nursing, Professional") OR (MH "Obstetric Nursing") OR (MH "Pediatric Nursing") OR (MH "Nurse Midwifery") 3. S1 OR S2 4. (AB((home N3 nursing) OR (health* N1 visitor*) OR midwi* OR (health* N1 professional*) OR (health* N1 personnel) OR (health* N1 worker*) OR (health* N1 provider*) OR nurse*))) OR (TI((home N3 nursing) OR (health* N1 visitor*) OR midwi* OR (health* N1 professional*) OR (health* N1 personnel) OR (health* N1 worker*) OR (health* N1 provider*) OR nurse*))) 5. S1 OR S2 OR S3 OR S4 6. (MH "Postnatal Period") OR (MH "Postnatal Care") 7. (AB(postnatal* OR "post natal*" OR postpartum OR "post partum" OR puerperi*)) OR (TI(postnatal* OR "post natal*" OR postpartum OR "post partum" OR puerperi*)) 8. S6 OR S7 9. S5 AND S8 10. municipal* OR local* OR district* OR communit* OR communal* OR rural* OR home* 11. S9 AND S10 12. (MH "Attitude of Health Personnel") OR (MH "Midwife Attitudes") OR (MH "Nurse Attitudes") OR (MH "Employee Attitudes") 13. (AB((perspective* OR experience* OR view* OR perce* OR attitude*) N10 ((health* N1 visitor*) OR midwi* OR (health* N1 professional*) OR (health* N1 personnel) OR (health* N1 worker*) OR (health* N1 provider*) OR nurse?)))) OR (TI((perspective* OR experience* OR view* OR perce* OR attitude*) N10 ((health* N1 visitor*) OR midwi* OR (health* N1 professional*) OR (health* N1 personnel) OR (health* N1 worker*) OR (health* N1 provider*) OR nurse?)))) 14. S12 OR S13 15. S11 AND S14 16. MH ("Qualitative Studies+" OR "Thematic Analysis" OR "Content Analysis" OR "Focus Groups" OR "Ethnographic Research" OR "Field Studies" OR "Narratives+" OR "Multimethod Studies" OR "Observational Methods+" OR "Audiorecording" OR "Semi-Structured Interview" OR "Unstructured Interview") OR TI (metaethno* OR "meta-synthes*" OR metasynthes* OR “thematic analys*” OR “content analys*” OR “focus group*” OR ethnograph* OR ethnograf* OR etnograf* OR “field stud*” OR phenomenolog* OR hermeneutic* OR narration* OR narrative OR “qualitative stud*” OR “qualitative analys*” OR “qualitative research*” OR “qualitative method*” OR multimethodolog* OR “mixed method*” OR observation* OR “grounded theory” OR “audio recording*” OR “tape recording*” OR audiotape* OR ((“semi-structured” OR semistructured OR unstructured OR informal OR “in-depth” OR indepth OR “face-to-face” OR structured OR guide*) AND (interview* OR discussion* OR questionnaire*))) OR AB (metaethno* OR "meta-synthes*" OR metasynthes* OR “thematic analys*” OR “content analys*” OR “focus group*” OR ethnograph* OR ethnograf* OR etnograf* OR “field stud*” OR phenomenolog* OR hermeneutic* OR narration* OR narrative OR “qualitative stud*” OR “qualitative analys*” OR “qualitative research*” OR “qualitative method*” OR multimethodolog* OR “mixed method*” OR observation* OR “grounded theory” OR “audio recording*” OR “tape recording*” OR audiotape* OR ((“semi-structured” OR semistructured OR unstructured OR informal OR “in-depth” OR indepth OR “face-to-face” OR structured OR guide*) AND (interview* OR discussion* OR questionnaire*))) 17. S15 AND S16 18. S15 AND S16 19. S15 AND S16 |
| Number of hits | 234 |
| Comments | Limiters/Expanders  Exclude MEDLINE records  Narrow by Language: - danish  Narrow by Language: - english |

| Database/source | **Ovid MEDLINE**(R) ALL <1946 to June 02, 2022> |
| --- | --- |
| Date of search | 3 June 2022 |
| Search history or procedure | 1. Midwifery/ or health personnel/ or nurses/ or nurse midwives/ or nurses, community health/ or nurses/ or community health nursing/ or home health nursing/ or home care services/ or community health nursing/ or home care nursing/ or postnatal care/ 181295 2. ((home adj4 nurs*) or (health* adj2 visitor*) or midwi* or (health* adj2 professional*) or (health* adj2 personnel) or (health* adj2 worker*) or (health* adj2 provider*) or nurse?).ab,ti,kf. 567413 3. 1 or 2 653900 4. (postnatal* or post natal* or postpartum or post partum or puerperi*).ab,ti,kf. 201573 5. (municipal* or local* or district* or communit* or communal* or rural* or home*).ab,ti,kf,hw.3110291 6. attitude/ or "attitude of health personnel"/ 180391 7. ((perspective* or experience* or view* or perce* or attitude*) adj11 ((health* adj2 visitor*) or midwi* or (health* adj2 professional*) or (health* adj2 personnel) or (health* adj2 worker*) or (health* adj2 provider*) or nurse?)).ab,ti,kf,hw. 193892 8. 6 or 7 243591 9. 3 and 4 and 5 and 8 642 10. limit 9 to "qualitative (maximizes sensitivity)" 537 11. (thematic analys* or content analys* or focus group? or ethnograph* or ethnograf* or etnograf* or field stud* or phenomenolog* or hermeneutic* or narration* or narrative? or qualitative stud* or qualitative analys* or qualitative research* or qualitative method* or multimethodolog* or multi methodolog* or mixed method* or observation* or grounded theory or audio recording* or tape recording* or audiotape* or ((semi-structure* or semistructure* or unstructure* or informal or "in-depth" or indepth or "face-to-face" or structured or guide*) and (interview* or discussion* or questionnaire* or metaethno* or meta-synthes* or metasynthes*))).ti,ab,kf,hw. 1529821 12. 9 and 11 391 13. 10 or 12 574 14. remove duplicates from 13 573 |
| Number of hits | 573 |
| Comments |  |

| Database/source | **Embase** <1974 to 2022 June 02> |
| --- | --- |
| Date of search | 3 June 2022 |
| Search history or procedure | 1. midwife/ or nurse midwife/ or nurse practitioner/ or nurse specialist/ or advanced practice nurse/ or family nurse practitioner/ or pediatric nurse practitioner/ or nurse/ or practical nurse/ or registered nurse/ or health care personnel/ or postnatal care/ 394080 2. ((home adj4 nurs*) or (health* adj2 visitor*) or midwi* or (health* adj2 professional*) or (health* adj2 personnel) or (health* adj2 worker*) or (health* adj2 provider*) or nurse?).ab,ti,kf. 698360 3. 1 or 2 839840 4. puerperium/ or postnatal care/ 51828 5. (postnatal* or post natal* or postpartum or post partum or puerperi*).ab,ti,kf.   254344   1. 4 or 5 266283 2. (municipal* or local* or district* or communit* or communal* or rural* or home*).ab,ti,kf,hw. 3930617 3. attitude/ or health personnel attitude/ or midwife attitude/ or nurse attitude/   188923   1. ((perspective* or experience* or view* or perce* or attitude*) adj11 ((health* adj2 visitor*) or midwi* or (health* adj2 professional*) or (health* adj2 personnel) or (health* adj2 worker*) or (health* adj2 provider*) or nurse?)).ab,ti,kf,hw.   201241   1. 8 or 9 265468 2. 3 and 6 and 7 and 10 832 3. limit 11 to "qualitative (maximizes sensitivity)" 566 4. (thematic analys* or content analys* or focus group? or ethnograph* or ethnograf* or etnograf* or field stud* or phenomenolog* or hermeneutic* or narration* or narrative? or qualitative stud* or qualitative analys* or qualitative research* or qualitative method* or multimethodolog* or multi methodolog* or mixed method* or observation* or grounded theory or audio recording* or tape recording* or audiotape* or ((semi-structure* or semistructure* or unstructure* or informal or "in-depth" or indepth or "face-to-face" or structured or guide*) and (interview* or discussion* or questionnaire* or metaethno* or meta-synthes* or metasynthes*))).ti,ab,kf,hw. 2005734 5. 11 and 13 478 6. 12 or 14 613 7. remove duplicates from 15 604 8. limit 16 to embase 250 |
| Number of hits | 250 |
| Comments |  |

| Database/source | **BRITISH NURSING INDEX** |
| --- | --- |
| Date of search | 3 June 2022 |
| Search history or procedure | (MAINSUBJECT.EXACT("Midwifery") OR MAINSUBJECT.EXACT("Certified nurse-midwives") OR MAINSUBJECT.EXACT("Nurse practitioners") OR MAINSUBJECT.EXACT("Nurse specialists") OR MAINSUBJECT.EXACT("Advanced practice nurses") OR MAINSUBJECT.EXACT("Visiting nurses") OR MAINSUBJECT.EXACT("Nurses") OR MAINSUBJECT.EXACT("Medical personnel") OR MAINSUBJECT.EXACT("Nursing care") OR MAINSUBJECT.EXACT("Maternal child nursing") OR MAINSUBJECT.EXACT("Maternal & child health") OR noft((home NEAR/3 nursing) OR (health* NEAR/1 visitor*) OR midwi* OR (health* NEAR/1 professional*) OR (health* NEAR/1 personnel) OR (health* NEAR/1 worker*) OR (health* NEAR/1 provider*) OR nurse?)) AND (MAINSUBJECT.EXACT("Postpartum period") OR noft(postnatal* OR ("post natal") OR postpartum OR "post partum" OR puerperi*)) AND noft(municipal* OR local* OR district* OR communit* OR communal* OR rural* OR home*) AND ((MAINSUBJECT.EXACT("Employee attitude") OR noft((perspective* OR experience* OR view* OR perce* OR attitude*) NEAR/10 ((health* NEAR/1 visitor*) OR midwi* OR (health* NEAR/1 professional*) OR (health* NEAR/1 personnel) OR (health* NEAR/1 worker*) OR (health* NEAR/1 provider*) OR nurse?)))) AND (MAINSUBJECT.EXACT("Qualitative research") OR MAINSUBJECT.EXACT("Grounded theory") OR MAINSUBJECT.EXACT("Phenomenological research") OR MAINSUBJECT.EXACT("Phenomenology") OR (MAINSUBJECT.EXACT("Action research") OR MAINSUBJECT.EXACT("Interviews") OR MAINSUBJECT.EXACT("Field study") OR MAINSUBJECT.EXACT("Audio recordings") OR MAINSUBJECT.EXACT("Storytelling") OR MAINSUBJECT.EXACT("Content analysis") OR MAINSUBJECT.EXACT("Ethnography") OR MAINSUBJECT.EXACT("Narratives") OR noft("thematic analys*" OR "content analys*" OR "focus group*" OR ethnogra* OR etnograf* OR "field student*" OR "field studies" OR "field study" OR "field studying" OR phenomenolog* OR hermeneutic* OR narration* OR narrative OR "qualitative studies" OR "qualitative study" OR "qualitative analy*" OR "qualitative research*" OR "qualitative method*" OR multimethodolog* OR "multi methodolog*" OR "mixed method" OR "mixed methods" OR observation* OR "grounded theory" OR "audio recording*" OR audiorecording* OR "tape recording*" OR taperecording* OR audiotape* OR (("semi-structured" OR semistructured OR unstructured OR informal OR "in-depth" OR indepth OR "face-to-face" OR structured OR guide*) NEAR/2 (interview* OR discussion* OR questionnaire*)) OR metaethno* OR "meta-synthes*" OR metasynthes*)))= 124 results |
| Number of hits | 124 |
| Comments |  |

| Database/source | APA **PsycInfo** <1806 to May Week 4 2022> |
| --- | --- |
| Date of search | 3 June 2022 |
| Search history or procedure | 1. midwifery/ 1612 2. health personnel/ 19261 3. nurses/ or public health service nurses/ 31091 4. ((home adj4 nurs*) or (health* adj2 visitor*) or midwi* or (health* adj2 professional*) or (health* adj2 personnel) or (health* adj2 worker*) or (health* adj2 provider*) or nurse?).ab,ti,id. 165005 5. 1 or 2 or 3 or 4 172281 6. postnatal period/ 5768 7. (postnatal* or post natal* or postpartum or post partum or puerperi*).ab,ti,id. 35598 8. 6 or 7 36103 9. (municipal* or local* or district* or communit* or communal* or rural* or home*).ab,ti,id,hw. 671052 10. exp Attitudes/ or exp Health Personnel Attitudes/ 417723 11. ((perspective* or experience* or view* or perce* or attitude*) adj11 ((health* adj2 visitor*) or midwi* or (health* adj2 professional*) or (health* adj2 personnel) or (health* adj2 worker*) or (health* adj2 provider*) or nurse?)).ab,ti,hw. 47124 12. ((perspective* or experience* or view* or perce* or attitude*) and ((health* adj2 visitor*) or midwi* or (health* adj2 professional*) or (health* adj2 personnel) or (health* adj2 worker*) or (health* adj2 provider*) or nurse?)).id. 10388 13. 10 or 11 or 12 439198 14. qualitative methods/ or exp focus group/ or grounded theory/ or interpretative phenomenological analysis/ or narrative analysis/ or semi-structured interview/ or thematic analysis/ or phenomenology/ or qualitative measures/ 34870 15. hermeneutics/ 2362 16. (thematic analys* or content analys* or focus group? or ethnograph* or ethnograf* or etnograf* or field stud* or phenomenolog* or hermeneutic* or narration* or narrative? or qualitative stud* or qualitative analys* or qualitative research* or qualitative method* or multimethodolog* or multi methodolog* or mixed method* or observation* or grounded theory or audio recording* or tape recording* or audiotape* or ((semi-structure* or semistructure* or unstructure* or informal or "in-depth" or indepth or "face-to-face" or structured or guide*) and (interview* or discussion* or questionnaire* or metaethno* or meta-synthes* or metasynthes*))).ti,ab,id. 583248 17. 14 or 15 or 16 585177 18. 5 and 8 and 9 and 13 and 17 99 |
| Number of hits | 99 |
| Comments |  |

| Database/source | **Web of Science** |
| --- | --- |
| Date of search | 3 June 2022 |
| Search history or procedure | 1. TS=(«focus group$» OR »grounded theory” OR ”phenomenolog*analys” OR ”semi-structured interview*” OR ”thematic analys*” OR phenomenology OR ”qualitative measures” OR hermeneutic OR ”content analys*” OR ethnograph* OR ethnograf* OR ”field stud*” OR narration* OR narrative$ OR ”qualitative stud*” OR “qualitative analys*” OR “qualitative research*” 2. TS=((home NEAR/3 nursing) OR (health* NEAR/1 visitor*) OR midwi* OR (health* NEAR/1 professional*) OR (health* NEAR/1 personnel) OR (health* NEAR/1 worker*) OR (health* NEAR/1 provider*) OR nurse*) 3. TS=(postnatal* or “postnatal*” or postpartum or “post partum” or puerperi*) 4. TS=(municipal* OR local* OR district* OR communit* OR communal* OR rural* OR home*) 5. TS=((perspective* OR experience* OR view* OR perception* OR perceive* OR attitude*) NEAR/10 ((health*NEAR/1 visitor*) OR (health*NEAR/1 professional*) OR (health*NEAR/1 personnel*) OR (health*NEAR/1 worker*) OR (health*NEAR/1 provider*) OR nurse$ OR midwi*)) 6. #5 AND #4 AND #3 AND #2 AND #1 7. #5 AND #4 AND #3 AND #2 AND #1 and English or Danish (Languages) |
| Number of hits | 292 |
| Comments |  |
